# Quantitative MR Markers in Non-Myelopathic Spinal Cord Compression: A Narrative Review

**DOI:** 10.3390/jcm11092301

**Published:** 2022-04-20

**Authors:** Jan Valošek, Petr Bednařík, Miloš Keřkovský, Petr Hluštík, Josef Bednařík, Alena Svatkova

**Affiliations:** 1 Department of Neurology, Faculty of Medicine and Dentistry, Palacký University Olomouc, 779 00 Olomouc, Czech Republic; jan.valosek@upol.cz (J.V.); phlustik@upol.cz (P.H.); 2 Department of Radiology, Faculty of Medicine and Dentistry, Palacký University Olomouc, 779 00 Olomouc, Czech Republic; 3 Department of Biomedical Engineering, University Hospital Olomouc, 779 00 Olomouc, Czech Republic; 4 Danish Research Centre for Magnetic Resonance, Centre for Functional and Diagnostic Imaging and Research, Copenhagen University Hospital Amager and Hvidovre, 2650 Hvidovre, Denmark; petrb@drcmr.dk; 5 Department of Radiology, Centre for Functional and Diagnostic Imaging and Research, Copenhagen University Hospital Amager and Hvidovre, 2650 Hvidovre, Denmark; 6 Faculty of Medicine, Masaryk University, 625 00 Brno, Czech Republic; kerkovsky.milos@fnbrno.cz (M.K.); bednarik.josef@fnbrno.cz (J.B.); 7 Department of Radiology and Nuclear Medicine, University Hospital Brno, 625 00 Brno, Czech Republic; 8 Department of Neurology, University Hospital Olomouc, 779 00 Olomouc, Czech Republic; 9 Department of Neurology, University Hospital Brno, 625 00 Brno, Czech Republic; 10 Central European Institute of Technology, Masaryk University, 625 00 Brno, Czech Republic; 11 Department of Medicine III, Clinical Division of Endocrinology and Metabolism, Medical University of Vienna, 1090 Vienna, Austria

**Keywords:** non-myelopathic cervical spinal cord compression, degenerative cervical myelopathy, diffusion magnetic resonance imaging, quantitative magnetic resonance imaging

## Abstract

Degenerative spinal cord compression is a frequent pathological condition with increasing prevalence throughout aging. Initial non-myelopathic cervical spinal cord compression (NMDC) might progress over time into potentially irreversible degenerative cervical myelopathy (DCM). While quantitative MRI (qMRI) techniques demonstrated the ability to depict intrinsic tissue properties, longitudinal in-vivo biomarkers to identify NMDC patients who will eventually develop DCM are still missing. Thus, we aim to review the ability of qMRI techniques (such as diffusion MRI, diffusion tensor imaging (DTI), magnetization transfer (MT) imaging, and magnetic resonance spectroscopy (^1^H-MRS)) to serve as prognostic markers in NMDC. While DTI in NMDC patients consistently detected lower fractional anisotropy and higher mean diffusivity at compressed levels, caused by demyelination and axonal injury, MT and ^1^H-MRS, along with advanced and tract-specific diffusion MRI, recently revealed microstructural alterations, also rostrally pointing to Wallerian degeneration. Recent studies also disclosed a significant relationship between microstructural damage and functional deficits, as assessed by qMRI and electrophysiology, respectively. Thus, tract-specific qMRI, in combination with electrophysiology, critically extends our understanding of the underlying pathophysiology of degenerative spinal cord compression and may provide predictive markers of DCM development for accurate patient management. However, the prognostic value must be validated in longitudinal studies.

## 1. Introduction

The resilience of the cervical spinal cord (SC) to incipient compressive changes, such as intervertebral disc bulging, herniation, or osteophyte formation, often leads to non-myelopathic degenerative cervical spinal cord compression (NMDC), a condition that precedes clinically manifested degenerative cervical myelopathy (DCM) [1,2,3,4]. Although the degenerative spinal cord compression (DSCC) occurs predominantly between the C4/5 and C6/7 cervical levels [5,6], secondary degenerative changes, such as axonal degeneration and demyelination, propagate remotely in both the superior and inferior directions, affecting levels above and below the compression and even leading to alterations in the brain [7,8,9]. The recent systematic review [10] showed that the prevalence of NMDC in the Caucasian population over 60 years is up to 39.7% and further increases with age [6,10,11]. Even though NMDC patients may only exhibit cervical axial pain and/or signs or symptoms of radiculopathy, without any signs or symptoms of clinical manifest myelopathy (Table 1), up to 23% of NMDC patients progress into symptomatic DCM during a follow-up of 44 months [3,12]. The current clinical guidelines [4,13] imply conservative clinical treatment in NMDC patients without symptoms of radiculopathy, whereas guidelines recommend the consideration of surgical intervention in NMDC patients with clinical and/or electrophysiological evidence of radiculopathy. Given the undeniable risks of decompressive surgery in 7–11% of patients [1], aging of the population worldwide, and substantially reduced quality of life in DCM patients [1,14], there is an urgent need to reliably identify NMDC patients with a higher risk of progression to irreversible DCM [13].

While previous reviews [1,7,15,16,17,18] focused on the epidemiology, pathophysiology, and assessment of DCM using structural MRI, diffusion tensor imaging (DTI) [19,20], and magnetic resonance spectroscopy (MRS) [20], so far, limited attention has been paid to NMDC patients. To date, a single systematic review by Smith et al. [10] covered the NMDC prevalence in structural MRI but did not discuss the benefits and pitfalls of quantitative MRI (qMRI) techniques, which provide crucial in-vivo insight into the pathophysiology of degenerative compression. Thus, our review aims to identify and discuss the potential of qMRI techniques to quantify NMDC alterations in vivo and determine the likeliness of progression to DCM. Due to the relatively limited number of qMRI studies in NMDC patients, DCM studies were also included to elaborate on their prospects in NMDC.

## 2. MRI in the Non-Myelopathic and Myelopathic Spinal Cord Compression

### 2.1. Structural MRI

Conventional clinical MRI is primarily acquired in the sagittal orientation to evaluate SC signal abnormalities, such as the presence of T2-w hyperintensities and T1-w hypointensities [31]. Subjectively-evaluated T2-w hyperintensities are still considered an important factor influencing decision-making for decompressive surgery [12], although their presence does not necessarily correspond with the clinical DCM signs and symptoms [32]. Intramedullary T2-w hyperintensities have, indeed, been reported in 58–85% of patients with clinically manifest DCM [33], whereas in NMDC inconsistently ranged between 2.3–24.6% [3,6,26,34]. T1-w hypointensities are associated with permanent SC injury [31], and they are relatively rare, occurring in 19–30% of DCM patients [32]; thus, their predictive value in NMDC patients is limited.

**Figure 1:**
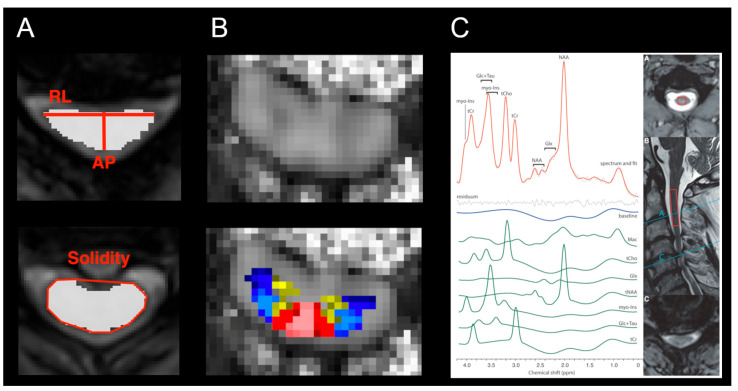
Quantitative MRI (qMRI) markers, derived using various qMRI methods. (**A**) Morphometric metrics measuring the degree of spinal cord compression, based on structural MRI. Upper panel shows compression ratio calculated as a ratio between the anteroposterior (AP) and transverse (RL) diameters, and lower panel shows solidity calculated as a ratio of cross-sectional area to the area of the smallest convex polygon surrounding all positive pixels in the image. Image courtesy of Magda Horáková. (**B**) Map of fractional anisotropy (FA), estimated using diffusion tensor imaging model from diffusion-weighted imaging data. Upper panel shows the FA map, and lower panel shows the FA map overlayed with probabilistic PAM50 atlas [41] of white and gray matter, allowing for tissue-specific analysis. Adapted with permission from Ref. [5] under creative common license; (**C**) single-voxel magnetic resonance spectroscopy (^1^H-MRS) measuring metabolic concentrations from above the compression level C2/3 (red box). Adapted with permission from Ref. [29] under creative common license.

In addition to the conventional clinical description of signal changes, sequences with a sufficient axial in-plane resolution below 1 mm and good contrast between white/gray matter (WM/GM) and cerebrospinal fluid (CSF) (typically 3D isotropic T1-w and 2D axial multi-echo gradient echo T2*-w sequences) allow for assessing morphometric metrics, in order to further validate the severity of compression. The cross-sectional area (i.e., area of the SC in the axial plane) of ≤70.1 mm^2^, and the compression ratio (i.e., the ratio between the anteroposterior diameter and the transverse diameter) of ≤0.4 distinguished NMDC patients who developed symptomatic DCM with sensitivities of 66.7 and 82.5, respectively, as well as specificities of 60.0 and 89.7, respectively [26]. Recently proposed morphometric metrics, reflecting SC flattening, indentation, and torsion (Figure 1A) [30,35], semi-automatically detected DSCC with the area under the curve of 0.947 (compared to expert raters); however, no morphometric metric distinguished between NMDC and DCM patients [30]. NMDC patients also showed an increased T2*-w WM/GM intensity ratio relative to healthy controls (HC) in a maximally compressed level (MCL) as well as rostrally and caudally [25].

**Figure 2:**
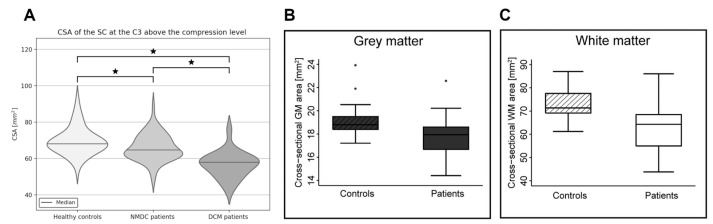
Significant reduction of the cross-sectional area (CSA) above the stenosis level. (**A**) Spinal cord (SC) CSA reduction at the C3 level, between NMDC and DCM patients, relative to healthy controls. Asterisk symbols (*) indicate significant difference between groups. Adapted with permission from Ref. [5] under creative common license; (**B**) Grey and (**C**) white CSA reduction at C2/3 level, between DCM patients and HC. Adapted with permission from Ref. [37] under creative common license.

SC volumetry adds to the compression metrics at MCL when assessing changes above and below the compression levels. So far, studies demonstrated a gradual reduction of SC, WM, and GM volumes at C2/3 above the compression level in DCM and NMDC, relative to HC (Figure 2) [5,29,36,37,38,39,40]. Exacerbation of alterations in DCM then NMDC points to more progressive Wallerian neurodegeneration and atrophy in DSCC [5,38]. A recent study also reported atrophy of SC, WM, and GM below MCL at the T11-L1 level in DCM patients, relative to HC, due to the trans-synaptic degeneration [40].

### 2.2. Microstructural Quantitative MRI

#### 2.2.1. Diffusion MRI

Diffusion magnetic resonance imaging (dMRI, or diffusion-weighted imaging, DWI) is sensitive to random water molecule movement within the tissue, which is restricted/hindered by myelination and axonal configuration [42]. Clinical dMRI has been used for the quantification of diffusion restriction or apparent diffusion coefficient caused, for example, by vasogenic edema due to acute ischemia [42]. The research applications rely on the fitting of diffusion models, which provide quantitative microstructural markers that are sensitive to different pathologies, such as axonal damage and demyelination [42]. The most commonly used diffusion model in the SC research is diffusion tensor imaging (DTI) [7,43,44]. DTI provides fractional anisotropy (FA) (Figure 1B), referring to the directional preference of diffusion, affected by the degree of myelination, axonal packing, axon size, coherence and co-linearity of fiber organization, mean diffusivity (MD) measuring the overall molecular diffusion rate, and axial (AD) and radial diffusivity (RD), referring to the degree of tissue edema, axonal damage, and demyelination, respectively [42,45]. However, DTI, as a single-compartment model, allows us to reconstruct only a primary diffusion direction and fails to estimate more complex WM fiber configurations [44]. Higher-order diffusion models, such as neurite orientation dispersion and density imaging (NODDI) [46,47,48,49,50,51], ball-and-sticks [5,27], and diffusion kurtosis imaging (DKI) [52,53], which overcome DTI’s limitation by modeling several tissue compartments, were recently translated from the brain to SC imaging, in order to provide more precise depiction of its complex microstructure.

##### Diffusion Tensor Imaging

Multiple studies [19,21,23,27,33,35,39,51,54,55,56,57,58,59,60,61,62,63,64,65,66,67,68,69,70] and reviews [19,20] covered DTI in symptomatic DCM patients, whereas only a few works are available in NMDC patients [5,21,23,24,25,26,27] (Table 2). One of the first 1.5T studies in NMDC patients compared the DTI metrics of 13 HC with 20 DCM and 32 NMDC patients and detected lower FA and higher MD at MCL in DCM, compared to NMDC patients, with lower FA and no significant MD deficits between NMDC patients and HC [21]. Outcomes were corroborated by the second study [23] on 37 DCM patients, 93 NMDC patients, and 71 HC with the same inclusion/exclusion criteria, although no comparison between NMDC patients and HC was provided. However, both studies employed a single ROI that covered the entire axial SC, and it is, thus, unclear whether the decreased FA was caused by a higher proportion of GM with naturally lower FA, compared to WM, or by actual WM damage. The first 3T NMDC DTI study [25] detected lower FA in the entire axial ROI at MCL in 20 NMDC patients, relative to 20 HC, and corroborated the previous 1.5T study [21], although it utilized a slightly distinct inclusion criteria, compared to the Czech studies [5,21,23] (Table 1). The additional column-specific analysis showed decreased FA in the ventral columns of NMDC patients [25]. A recent 3T tract-specific study detected lower FA and higher MD and RD at MCL in dorsal and lateral tracts in a large cohort of 103 NMDC and 21 DCM patients, compared to 60 HC with more profound alterations in DCM than NMDC [5]. In agreement with histopathology [1,71], which demonstrated malperfusion throughout the territory of compressed anterior spinal artery with restrained blood supplies in the lateral columns, anterior part of dorsal columns, and ventral GM horns, GM also showed a significant alteration, with higher MD, AD, and RD in both NMDC and DCM patients, relative to HC [5]. Another 1.5T study found lower FA and higher RD at MCL in the lateral corticospinal tracts in 16 DCM patients with clinical DCM symptoms, without evidence of SC damage on T2-w images, compared to 20 HC [72]; no changes were demonstrated in the remaining medial parts, further confirming demyelination in dorsal and lateral WM tracts [5,72].

Besides the direct changes at the stenosis level, studies have also focused on remote neurodegeneration above the compression level. DCM studies [5,36,37,39,40,65] consistently detected decreased FA and increased diffusivity measures at the C2/C3 level in the dorsal and lateral WM tracts, compared to HC, whereas only two DCM studies showed changes in rostral GM [5,37]. The recent NMDC study then detected similar changes at the C3 level, i.e., decreased FA and increased MD and RD in the dorsal and lateral tracts and increased diffusivity measures in GM between DCM and NMDC patients [5]. Thus, outcomes congruently demonstrated the remote rostral neurodegeneration of long lateral and dorsal WM tracts, as well as trans-synaptic degeneration of GM in symptomatic DCM patients, relative to HC. Incipient remote changes in NMDC patients, relative to HC, were not detectable by DTI [25] and were, so far, observed only using the multi-compartment ball-and-sticks model, emphasizing the need for further research and utilization of multi-compartment dMRI models [5].

Importantly, to date, only two DTI studies [24,26] examined NMDC patients longitudinally to monitor progression from NMDC to DCM, and both utilized entire axial ROI (i.e., tissue non-specific). The first study [26] monitored DCM development in 112 NMDC patients, in 3 years median follow-ups, using a 1.5T scanner, and found no predictive power of DTI. The second 3T study [24] followed-up 66 nonoperatively treated patients with DSCC, for an average follow-up of 1.4 years, and reported that 47 out of 66 patients showed stationary FA and MD.

##### High-Order Diffusion Models

While higher-order diffusion models have been frequently utilized in DCM studies [5,46,47,51,52,53,68], only a limited number of works also included NMDC patients [5,27].

**Figure 3:**
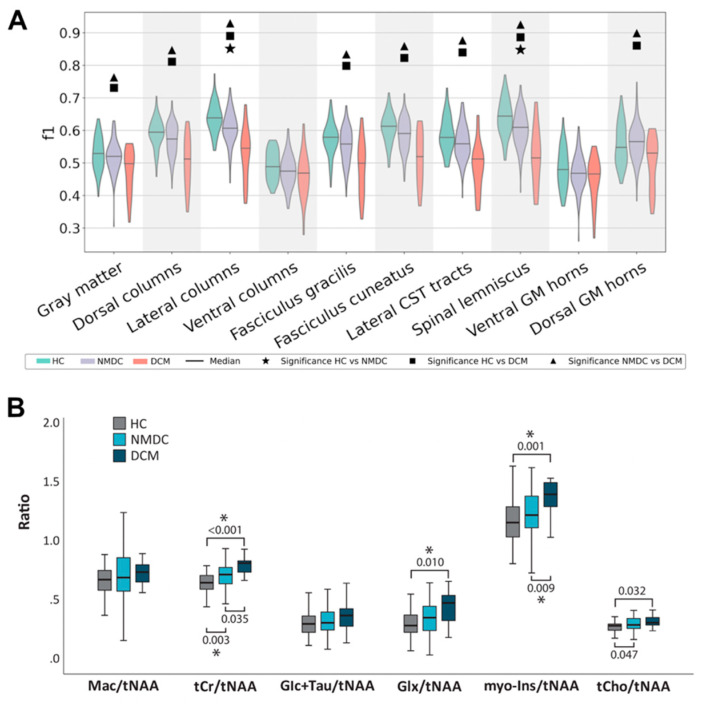
Group differences between NMDC and DCM patients, relative to healthy controls (HC). (**A**) Between-group differences in the f1 diffusion metric (i.e., primary partial volume fraction of the ball-and-sticks model) at C3, above the compression level. Adapted with permission from Ref. [5] under creative common license. (**B**) Between-group difference in neurometabolies ratios, gained from single-voxel magnetic resonance spectroscopy (^1^H-MRS) from above the compression level C2/3. Asterisk symbols (*) indicate significant difference between groups. Adapted with permission from Ref. [29] under creative common license.

Recently, a multi-shell diffusion protocol, with reduced field-of-view, allowed for the estimation of more complex diffusion models, such as the ball-and-sticks model [73], in addition to the DTI model [27]. The multi-compartment ball-and-sticks model describes diffusion by a single isotropic and several anisotropic compartments and better characterizes diffusion data than the single-compartment DTI model [74]. The ball-and-sticks model initially demonstrated sensitivity to subtle microstructural changes in both WM and GM in 33 NMDC patients, relative to 13 HC [27], and was thereafter used in a large cohort of 103 NMDC, 21 DCM patients, and 60 HC to delineate changes in dorsal and lateral tracts and GM between NMDC and HC at MCL, as well as rostrally (Figure 3A) [5]. Results suggest superior discriminant power of the multi-compartment ball-and-sticks model over DTI, when abnormalities were depicted in the f1 metric (i.e., the primary anisotropic volume fraction), which were not detectable by DTI [5].

Two retrospective studies [47,51], utilizing the three-compartment NODDI model [75], alongside DTI, to monitor surgical outcome in DCM patients, showed increased FA at MCL two weeks after surgery and increased intracellular volume fraction at MCL six months after surgery [47,51]. Conclusions indicate that neurite density damage in DCM patients might not be irreversible [47]. A concurrent DTI, NODDI, and DKI study demonstrated lower FA and DKI-FA and a higher DKI-MD, isotropic CSF volume fraction, and orientation dispersion index from the entire axial ROI at MCL in 48 DCM patients, relative to 36 HC [68]. The isotropic CSF volume fraction, FA, and DKI-FA also correlated with the recovery rate, calculated based on preoperative and three-month follow-up mJOA scales, indicating possible usage of these metrics as predictors in surgically-treated DCM patients [68].

So far, all published NODDI [47,51,68], DKI [52,53,68], and QSI [53] studies comprised solely of DCM patients, establishing a further need for the application of innovative dMRI techniques in NMDC patients.

##### Intravoxel Incoherent Motion Imaging

Intravoxel incoherent motion (IVIM) imaging measures the microscopic movement of water molecules caused by capillary perfusion, using a dMRI sequence with low b-values (≤300 mm^2^/s) to assess flowing blood fraction and pseudo-diffusion coefficient [76]. Pilot IVIM studies in the human SC at 7T in 6 HC [77] and at 3T in 2 DCM patients, along with 11 HC [78], depicted higher perfusion in GM, compared to WM in HC, and impaired perfusion in DCM patients at compression levels. However, interpretation is limited, due to the small sample size and possible influence of CSF pulsation [78]. IVIM imaging is a promising technique for future DCM and NMDC studies, as post-mortem studies showed that degenerative compression results in hypoperfusion and ischemia in specific WM/GM regions [1,71].

#### 2.2.2. Magnetization Transfer

Magnetization transfer (MT) imaging is based on the exchange of magnetization between the protons associated with free water and those linked with immobile macromolecules, such as proteins and lipids, and provides MT ratio (MTR) and MT saturation markers, which indirectly measure the myelination [79,80]. Martin et al. [25] reported decreased MTR extracted from the entire axial ROI in 20 NMDC subjects, compared to 20 HC above the compression (C1-C3), but not at MCL. Column-specific MTR analysis corroborated DTI when it demonstrated decreased MTR in ventral columns of NMDC subjects, relative to HC [25]. The same group also reported MTR, together with FA, cross-sectional area, and T2* WM/GM ratio, as useful measures within a composite score for monitoring 26 DCM patients in a 13.5 (mean) month follow-up and identified worsening in 11 DCM patients [35]. Another work then showed the predictive value of a combination of the preoperative MTR and shape analysis for surgery response and recovery in DCM patients [81]. Finally, a combination of MT imaging and dMRI was used to calculate myelin water fraction and axon volume fraction in 24 DCM patients, compared to 5 HC, and the results reported changes in axon volume fraction between groups in the fasciculus gracilis, fasciculus cuneatus, and lateral corticospinal tract [46].

#### 2.2.3. Magnetic Resonance Spectroscopy

Proton magnetic resonance spectroscopy (^1^H-MRS) quantifies the neurochemical profile within the spectroscopic volume of interest (i.e., spectroscopic voxel) and provides unique information about microstructural or metabolic pathophysiological processes that are inaccessible with conventional imaging methods (Figure 1C) [82,83]. SC ^1^H-MRS is challenged by its small transversal area, which is further diminished at the compression level. Therefore, ^1^H-MRS studies in DCM patients assessed the neurochemical profile only above the stenosis level and observed neurochemical changes rostrally to the compression, likely due to the Wallerian degeneration, which manifested as increased levels of total creatine (tCr)/total NAA (tNAA) [84,85,86,87] and total choline (tCho)/tNAA [85,86,88]. A recent ^1^H-MRS study in 47 HC, 60 NMDC, and 13 DCM patients showed, for the first time, increased tCr/tNAA and myo-Ins/tNAA ratios above the stenosis level in NMDC, relative to HC, pointing to neurochemical changes detectable in clinically silent subjects (Figure 3B) [29]. The high sensitivity of this study arises from superior accuracy in semi-LASER voxel localization [89], improved signal-to-noise ratio at high-field 3T scanner, and cardiac triggering, minimizing bias from surrounding tissue and cardiac pulsations [29].

Despite the degeneration of afferent tracts, which propagated the changes in DCM patients, up to the sensorimotor regions in the brain [8,9], the SC might display earlier alteration of the neurochemical profile and can be a more appropriate target to detect early markers in non-myelopathic compression. Several studies, indeed, suggested a potential predictive value of neurochemical markers when they showed a correlation between the severity of myelopathy symptoms (i.e., mJOA scale) and metabolite ratios [29,85,90].

#### 2.2.4. T1 and T2 Relaxometry

To date, T1 relaxometry, sensitive to myelination [45], provided contradictory outcomes, when it detected lower T1 times in 31 DCM patients at 1.5T at compression levels, compared to non-stenotic counterparts [91], but higher T1 times at 3T at compression levels in 22 DCM patients, compared to 10 HC [92]. Such opposite trends call for further harmonization of field strengths, imaging protocols, and inclusion criteria.

Thus far, 3T myelin water imaging, based on T2 relaxometry, demonstrated myelin content reduction in dorsal columns of 14 DCM patients with pathological somatosensory-evoked potentials [93], and the multicomponent-driven equilibrium steady-state estimation of myelin water fraction, as well as T1 and T2 times, provided a myelin imaging atlas of HC and setup framework for future studies [94].

#### 2.2.5. Functional MRI

Functional MRI (fMRI) measures the oscillations in neuronal activity by either a T2*-w sequence, sensitive to local magnetic field inhomogeneities related to blood oxygenation level-dependent effect, or arterial spin labeling sequences that utilize arterial blood as an endogenous tracer to measure cerebral blood flow [95]. Brain fMRI studies, indeed, revealed: remote changes in the activations of motor areas during finger-tapping tasks between DCM patients and HC [9,96], alterations of the sensorimotor network in resting-state fMRI in DCM patients [97], the relationship between severity of compression in DCM patients and activation volume in the motor cortex [98], and differences in brain activations in DCM patients with abnormal motor evoked potentials [99], suggesting that DSCC causes secondary brain changes. A single SC resting-state fMRI study showed neuronal activity changes in the GM horns of 18 DCM patients, relative to 25 HC, as well as an association of severity of myelopathy with neuronal activity response [100]. To date, no SC fMRI study has been performed in NMDC patients [101], further emphasizing the need to overcome the anatomy-related image distortions, low signal-to-noise ratio, and physiological movement artifacts [102], which limited fMRI use in patients with DSCC.

#### 2.2.6. Perfusion Weighted Imaging

Chronic DSCC reduces blood flow in spinal arteries and results in chronic SC ischemia in histological and animal models [1,71], which is a vital factor in DCM pathogenesis. While perfusion imaging methods, including dynamic susceptibility contrast (DSC), dynamic contrast-enhanced (DCE), and arterial spin labeling perfusion imaging, are commonly used in brain studies, there have been sparse applications in the SC [103,104,105]. A recent 3T study, in 22 DSCC patients with or without myelopathy, identified a significant relationship (*p* < 0.05) of DSC markers with the anteroposterior diameter and mJOA scale and suggested that the degree of ischemia and hypoxia correlates with compression severity and clinical status, respectively [103]. Another 1.5T DSC study, in 14 DCM patients, then showed improvement in the spinal perfusion after surgical decompression [104] and pseudo-continuous arterial spin labeling, which, unlike DSC and DCE, does not require an intravenous contrast agent, revealed secondary alteration of cerebral blood flow perfusion in DCM patients [105].

### 2.3. Spinal Cord MRI Data Acquisition and Processing

The SC is a small structure with anteroposterior and transverse diameters at the C2 level of 8.8 and 12.4 mm, respectively [106], which is placed in a bony spinal canal, surrounded by CSF, with variability in the magnetic susceptibilities. Thus, optimized acquisition protocols and dedicated analysis tools are required for accurate and reliable processing [79]. This need is further highlighted in patients with DSCC with altered anatomy.

#### 2.3.1. Data Acquisition

Sequences with sufficient in-plane resolution, signal-to-noise ratio, and clinically acceptable acquisition times of complete examination (under 30–40 min) are crucial for tissue-specific analysis. Generally, anisotropic resolution, on the order of 1 × 1 × 5 mm^3^, is recommended for dMRI and MT sequences, since the SC is a relatively homogenous structure in the superior-inferior direction, and higher slice thickness allows us to increase the signal-to-noise ratio and in-plane resolution [79,107]. Recently, the SC community released a prospectively harmonized *spine generic* acquisition protocol for 3T and 1.5T [107], allowing for multi-center studies [108]. Although higher field strength provides superior spatial resolution and signal-to-noise ratio, it introduces larger susceptibility artifacts and geometrical distortions, especially for dMRI sequences. Generally, dMRI sequences with reduced field-of-view are recommended over sequences with outer volume suppression to mitigate these artifacts [27,107,109]. Cardiac triggering might reduce pulsatile CSF flow and partial volume effect in dMRI [107,110] as well as ^1^H-MRS [29]. The acquisition of two dMRI sequences, with opposite phase-coding and usage of dedicated post-processing tools for correction of motion artifacts and geometrical distortions [111,112], were used across SC studies, even though these tools were primarily designed for the brain, and their usage for the SC is the subject of ongoing debate (https://forum.spinalcordmri.org/t/how-to-correct-for-distortions-in-spinal-cord-diffusion-mri-data/326, accessed on 15 January 2022). An increased signal-to-noise ratio of the 3T dMRI sequences also allows for acquiring multi-shell diffusion data with higher b-values, which is crucial for the fitting of multi-compartment diffusion models, such as NODDI, ball-and-sticks, or DKI [107]. Usually, high angular resolution diffusion imaging [113] sequences are employed, utilizing diffusion gradient sampling on several whole q-space spheres (i.e., multi-shell diffusion protocols) [114] and allowing for reliable estimation of the higher-order models.

The ^1^H-MRS sensitivity benefits from ultra-high fields [115,116], implementation of advanced shimming approaches minimizing anatomically determined pronounced B_0_-inhomogenety in the spinal canal [117], and prospective motion correction methods alleviating motion artifacts pronounced during longer acquisitions [118]. In addition, the automatization of ^1^H-MRS data acquisition, including automatic voxel placement, allows for shortening the scan and obtaining operator-independent data with the methodology previously implemented for the brain [119].

#### 2.3.2. Spinal Cord Data Processing

Analyses of the entire axial ROI in older NMDC works [21,23,24,26], which lack spatial resolution and did not allow for tracing the spatial origin of the observed microstructural changes, were overcome, thanks to probabilistic PAM50 atlas [41,120] and methods for minimizing of partial volume effect [41,121]. Atlas-based analysis was successfully used in several recent studies and revealed tissue-specific changes in both DCM and NMDC patients, as well as in patients with traumatic SC injury [5,25,65]. Alternative approaches for tract delineation are tractography [63,64,122], manually drawn ROIs [61,62,66], or the usage of tract-based spatial statistics (TBSS) approach [123]. However, tractography can suffer from inaccuracies caused by severe compression, and manually-defined ROIs are prone to user bias and take time to draw; thus, atlas-based approach is currently preferred [45,79].

**Figure 4:**
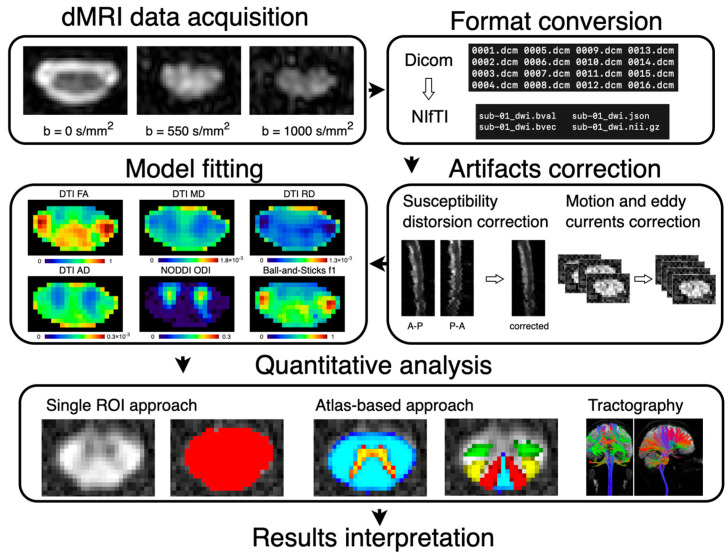
Typical dMRI workflow. dMRI data acquisition is followed by format conversion, usually from DICOM format, provided by the scanner, to NIfTI format [130], which is supported by many of neuroimaging tools. The subsequent processing pipeline typically includes correction of susceptibility-induced geometrical distortions, motion and eddy currents artifacts, and estimation of diffusion model(s). Final quantitative analysis can be done in various ways using a single region-of-interest (ROI) approach, atlas-based approach, or tractography. DTI, diffusion tensor imaging; FA, fractional anisotropy; MD, mean diffusivity; RD, radial diffusivity; AD, axial diffusivity; NODDI, neurite orientation dispersion and density imaging; ODI, orientation dispersion index; f1, primary partial volume fraction (anisotropic compartment of the ball-and-sticks model). The illustration of the tractography is reprinted with permission from Ref. [79]. Copyright, 2014, Elsevier.

The advent of the Spinal Cord Toolbox (SCT) [121] now allows for robust automatic segmentation of the SC and GM [124,125] and the processing of structural and qMRI images, as well as utilizing the probabilistic template and PAM50 atlas [41,120]. Alternative packages, such as FMRIB Software Library (FSL) [126], Statistical Parametric Mapping (SPM) software package [127], or JIM (http://www.xinapse.com, accessed on 15 January 2022), designed for brain analysis or dedicated libraries, such as Dipy [128] or LCmodel [129], for dMRI and MRS analysis, respectively, can also be used for SC data processing. Usually, a combination of tools is used to facilitate multimodal qMRI analysis; for example, SCT is utilized for automatic SC and GM segmentations, morphometric metrics extraction, and registration of PAM50 atlas, and it is supplemented by FSL or Dipy, which provide tools for fMRI analysis and the estimation of higher-order diffusion models. Note that anatomy altered by compression can negatively influence image acquisition and data processing, and it is, thus, necessary to perform quality checks, potential manual correction of segmentation, and adjustment of processing parameters (e.g., type of registration). Typical dMRI workflow is summarized in Figure 4.

### 2.4. Quantitative MRI in the Spinal Cord Compression and Correlations with Clinical Outcomes

A proper estimation of the relationship between qMRI markers and clinical status, assessed by mJOA scale [131] or electrophysiological measurements, is needed to gain insight into the clinical relevance of qMRI markers, prior to multicentric longitudinal trials. dMRI studies in DCM patients consistently reported significant correlations between the mJOA scale and FA (r > 0.45) [5,24] and MD (r = −0.32) [24], while MRS studies disclosed significant correlations between the mJOA scale and myo-Ins/tNAA (r = −0.67) [29], Cho/NAA (r > −0.44) [85,88], and NAA/Cr (r = 0.50) [85]. Nevertheless, the usage of the mJOA scale in NMDC patients is limited, since these patients are usually asymptomatic and, thus, without clinical deficits. T2-w signal intensity changes, electrophysiological abnormalities, and signs of radiculopathy were reported as predictors of progression from NMDC into DCM [3]; however, the subsequent studies did not find any association with DTI extracted from the entire axial SC ROI [21,26]. While Kadanka et al. [21], indeed, did not detect any significant difference in DTI markers from the entire axial SC in NMDC patients with and without electrophysiological abnormality, recent tissue-specific reports demonstrated a relationship between altered electrophysiology and DTI and ball-and-sticks metrics in both NMDC and DCM patients [5,60]. Diffusion metrics in the lateral motor and dorsal sensory tracts corresponded to alterations in motor and somatosensory-evoked potentials, and electromyography corresponded to diffusion metrics in GM [5,60]. Finally, Liu et al. [93] found a correlation between the decrease of myelin content in dorsal columns assessed by myelin water imaging and prolonged cortical somatosensory-evoked potential latencies in DCM patients.

## 3. Conclusions and Future Directions

While previous studies confirmed the SC microstructure alterations detected by qMRI in both NMDC and DCM patients, relative to HC, the results showed inconsistencies, due to distinctions in scanners’ field strength, acquisition protocols, and data post-processing. Additionally, unification of the inclusion criteria is particularly needed for NMDC individuals, as some studies include only those without radiculopathy [25], while others also incorporated NMDC subjects with radiculopathy [5,21,23].

To date, DTI studies at 1.5T and 3T consistently detected lower FA and higher MD at MCL in NMDC and DCM patients, relative to HC, with more progressive changes in DCM, compared to NMDC. These changes are likely caused by edema, deficits in the degree of myelination, axonal packing, and axon size. Some also found RD and MTR abnormalities pointing to demyelination [5,25,72] and AD alteration, due to axonal injury, as the primary alteration at MCL [5]. Rostral secondary changes in DCM patients presented as lower FA and higher diffusivity measures in dorsal columns and lateral corticospinal tracts, and alterations in ^1^H-MRS ratios at the C2/3 level point to remote Wallerian degeneration above the compression level [5,29,36,37,39,40,65,88,132], accompanied by the SC, WM, and GM volumes reduction [5,29,36,37,38,39,40]. Subtle remote changes at the C2/3 level between NMDC and HC were unraveled by the multi-compartment ball-and-sticks diffusion model, ^1^H-MRS, and MTR [5,25,29]. Moreover, brain fMRI and ^1^H-MRS studies in DCM patients showed secondary changes, even in the brain, suggesting alterations in neuronal activations and brain plasticity caused by DSCC [8,9]. Existing studies also showed the relationship between clinical impairments, assessed by clinical scales and microstructural degeneration, measured using qMRI [5,24,29,62,85,88]. Several works also provided evidence of the relationship between functional impairments, measured using electrophysiology and tract-specific qMRI metrics [5,60,93].

The widespread availability of 3T scanners in the clinical practice also further emphasizes the need to harmonize protocols across scanners and vendors, in order to estimate normative values, which was, so far, limited by the usage of different sequences and acquisition parameters. Indeed, the release of the *spine generic* acquisition protocol [107] provided a critical step forward for the upcoming longitudinal multicentric studies, with the promise of normative quantitative values. The 3T protocols, which minimize image artifacts, while benefitting from increased signal-to-noise ratio, compared to lower fields, are essential for methods such as dMRI and ^1^H-MRS [79]. High in-plane resolution of recent dMRI and MT sequences [5,25,40,110] allowed for tissue- and tract-specific analysis. Lastly, pilot studies at 7T showed promising results for future research that might further increase our understanding of metabolic and microstructural damage, yet the utilization will require further sequence development and usage of dedicated coils. 

In conclusion, while high-resolution 3T qMRI, with tissue- and tract-specific analysis, supplemented by electrophysiological measures and clinical scales, indeed showed ongoing microstructure alterations, even in NMDC patients, longitudinal and multicentric studies with optimized protocols are critical for future NMDC research. The application of qMRI, as a potential predictor of progression from NMDC to clinically manifested DCM, must be further verified by an estimation of the normative values for clinical practice; however, such a goal requires the harmonization of SC protocols across scanners and vendors.

## Figures and Tables

**Table 1:** Nomenclature and definitions of non-myelopathic spinal cord compression across studies.

Study	Nomenclature	Definition
	Original Articles	
Bednarik et al., 2004 [2], 2008 [3]	Pre-symptomatic spondylotic cervical cord compression (P-SCCC)	MR signs of DSCC (spondylotic or discogenic) and axial cervical pain or clinical signs and/or symptoms of radiculopathy, but no clinical signs of myelopathy (mJOA ≥ 16; note—mJOA decreased, due to radiculopathy)
Keřkovský et al., 2012 [21]	Asymptomatic spondylotic cervical cord encroachment (SCCE)	MR signs of DSCC and cervical pain and/or symptoms/signs of cervical radiculopathy, but without symptoms/signs of cervical spondylotic myelopathy (mJOA = 18)
Adamova et al., 2015 [22]	Asymptomatic spondylotic cervical cord compression (ASCCC)	No detailed description (study focused on prevalence of ASCCC in patients with clinically symptomatic lumbar spinal stenosis) (mJOA not reported)
Kovalova et al., 2016 [6]	Non-myelopathic spondylotic cervical cord compression (NMSCCC)	MR signs of DSCC and possible presence of radiculopathy, but no myelopathic signs (mJOA not reported)
Keřkovský et al., 2017 [23]	Asymptomatic degenerative cervical cord compression (ADCCC)	MR finding of DSCC and various clinical signs of cervical spine degenerative disease (cervical pain and radiculopathy), but no signs or symptoms of DCM (mJOA = 18)
Ellingson et al., 2018 [24]	Asymptomatic cervical stenosis	No neurological symptomatology (mJOA = 18), but complaints of neck pain
Martin et al., 2018 [25]	Asymptomatic spinal cord compression (ASCC)	MR finding of DSCC, but an absence of any neurological symptoms and signs; neck pain was not considered a neurological symptom (mJOA = 18)
Kadanka Jr. et al., 2017 [26], Labounek et al., 2020 [27]	Non-myelopathic degenerative cervical cord compression (NMDCCC)	MR signs of DSCC, but an absence of any myelopathic signs, possible presence of axial pain, symptoms or signs of upper extremity monoradiculopathy, or completely asymptomatic individuals (mJOA not reported)
Kadanka Jr. et al., 2021 [28]	Non-myelopathic degenerative cervical cord compression (NMDCC)	MR signs of DSCC and presence of maximally one clinical myelopathic symptom, but no clinical myelopathic signs (mJOA ≥ 17)
Valošek et al., 2021 [5], Horak et al., 2021 [29], Horakova et al., 2022 [30]	Non-myelopathic degenerative cervical spinal cord compression (NMDC)	MR signs of DSCC with or without radiculopathy and electrophysiological changes, but without myelopathic symptoms/signs (mJOA = 18)
	Reviews	
Wilson et al., 2013 [12]	Non-myelopathic patients with cervical stenosis	Review—no single definition
Witiw et al., 2018 [11]	Asymptomatic cervical spinal cord compression (CSCC)	Review—no single definition
Smith et al., 2020 [10]	Asymptomatic spinal cord compression (ASCC)	Review—no single definition
Badhiwala et al., 2020 [1]	Cervical spinal cord compression without myelopathy	Review—MR signs of DSCC, absence of any myelopathic signs, and clinical radiculopathy with or without electrophysiological changes or no signs of symptoms of radiculopathy (mJOA = 18)

DSCC, degenerative spinal cord compression; mJOA, modified Japanese Orthopaedic Association scale; MR, magnetic resonance.

**Table 2:** List of studies comprising of patients with non-myelopathic/asymptomatic spinal cord compression utilizing qMRI techniques. Studies are ordered chronologically.

Study	Cohort	Field Strength, Voxel Size, qMRI Technique, ROI	Key Results	Conclusion/Interpretation
Keřkovský et al., 2012 [21]	32 NMDC patients (mJOA = 18)	1.5T	Lower FA and higher MD at MCL in DCM, compared to NMDC	DTI showed potential to discriminate between NMDC and symptomatic DCM patients
	20 DCM patients (mJOA < 18)	1.25 × 1.25 × 4 mm^3^	Lower FA, no MD change at MCL in NMDC, relative to HC	Differences between NMDC and HC could be caused by demyelination, but potentially also by WM/GM mixing
	13 HC	DTI (FA, MD), entire axial SC		There was no difference in any of the DTI parameters for subsets of patients with and without electrophysiological abnormality
Keřkovský et al., 2017 [23]	93 NMDC patients (mJOA = 18)	1.5T	Lower FA and increased MD at MCL in DCM, compared to NMDC	DTI showed differences in FA and MD between NMDC and symptomatic DCM patients
	37 DCM patients (mJOA < 18)	1.25 × 1.25 × 4 mm^3^		No differences between NMDC and HC reported
	71 HC	DTI (FA, MD), entire axial SC		
Kadanka et al., 2017 [26]	40 NMDC patients (mJOA not reported)	1.5T	DTI parameters showed no significant predictive power in longitudinal follow-up	The development of DCM was associated with several parameters, such as radiculopathy or electrophysiological measures
	72 subjects with cervical radiculopathy or cervical pain (mJOA not reported)	1.25 × 1.25 × 4 mm^3^		DTI parameters showed no significant predictive power
		DTI (FA, MD), entire axial SC		
Martin et al., 2018 [25]	20 NMDC patients (mJOA = 18)	3T	Lower FA at MCL in entire axial ROI and ventral columns in NMDC, compared to HC	Changes in FA, MTR, and T2*WI WM/GM intensity point to demyelination and axonal injury as predominant pathogenic mechanisms in NMDC patients
	20 HC	1.25 × 1.25 × 5 mm^3^ (DWI); 1 × 1 × 5 mm^3^ (MT)	Lower MTR in the rostral region (C1-C3) and ventral columns in NMDC, compared to HC	Changes were observed at MCL, but also rostrally and caudally
		DTI (FA), MT (MTR) and T2*WI WM/GM, entire axial ROI and WM columns and GM	Higher T2*WI WM/GM at MCL and in rostral and caudal regions in NMDC compared to controls	
Ellingson et al., 2018 [24]	18 NMDC patients (mJOA = 18)	3T	Most patients (47 from 66) showed stationary longitudinal DTI measurements	DTI metrics correlated with neurological impairments, assessed by the mJOA scale, and may be valuable predictors of neurological status
	48 patients with clinical symptoms (mJOA < 18)	1.1 × 1.1 × 4–5 mm^3^	Pooled FA and MD at MCL from all patients and all time points showed correlation with mJOA scale	
		DTI (FA, MD), entire axial SC		
Labounek et al., 2020 [27]	33 NMDC patients (divided into two groups—mild and severe compression)	3T	Lower MD in WM in NMDC with mild compression, compared to HC	DTI and ball-and-sticks models demonstrated differences between HC and NMDC patients in both WM and GM
	13 HC	0.65 × 0.65 × 3.00 mm^3^ (interpolated)	Higher MD and d in GM in NMDC with severe compression, relative to HC	Optimized multi-shell dMRI protocol, with reduced field-of-view, outperformed clinically used single-shell protocol
		DTI (FA, MD) and ball-and-sticks model (f1, d), WM–GM difference, and “heuristic” parameters derived from these metrics, WM, and GM	Lower WM–GM difference for MD and d in NMDC with mild and severe compression, compared to HC	
			Difference in several “heuristic” parameters derived from FA, MD, f1, and d between groups, see the study [27] for details	
Valošek et al., 2021 [5]	103 NMDC patients (mJOA = 18)	3T	Lower FA and f1 and higher MD, AD, RD, and d in NMDC and DCM, compared to HC, with more severe changes in DCM, compared to NMDC	Compression primary affected lateral and dorsal white matter tracts and gray matter, pointing to demyelination and trans-synaptic degeneration
	21 DCM patients (mJOA < 18)	0.65 × 0.65 × 3.00 mm^3^ (interpolated)	Changes were detected predominantly in dorsal and lateral tracts and GM at MCL and rostrally at the C3 level	Above the compression changes suggest Wallerian degeneration
	60 HC	DTI (FA, MD, AD, RD) and ball-and-sticks models (f1, d), WM columns and tracts, and GM regions	DCM patients showed changes also in the ventral columns, compared to HC	Changes were more profound in DCM, compared to NMDC and HC, suggesting progressive changes in patients with compression over time
			dMRI changes correlated with the mJOA scale and reflected electrophysiological findings	Ball-and-sticks model showed changes not detected by DTI model
Horak et al., 2021 [29]	60 NMDC patients (mJOA = 18)	3T	Increased total creatin/tNAA ratio in NMDC and DCM, relative to HC	^1^H-MRS revealed neurochemical changes at the above the compression level C2/3 in both DCM and NMDC, compared to HC
	13 DCM patients (mJOA < 18)	8 × 9 × 45 mm^3^ (single MRS voxel)	Changed myo-inositol/tNAA and glutamate + glutamine/tNAA ratios in DCM, compared to HC	Neurochemical changes suggest demyelination and Wallerian degeneration
	47 HC	^1^H-MRS	myo-inositol/tNAA ratio in DCM patients correlated with the mJOA scale	
	102 NMDC (mJOA = 18)	1.5T and 3T	Logistic model combining compression ratio, cross-sectional area, solidity, and torsion detected compression with AUC = 0.947 (compared to expert raters)	The semi-automated method demonstrated outstanding compression detection, with better inter-trial variability, compared to manual raters
Horakova et al., 2022 [30]	16 DCM (mJOA < 18)	0.60 × 0.60 × 4.0 mm^3^ (1.5T) 0.35 × 0.35 × 2.5 mm^3^ (3T – interpolated)	The inter-trial variability (1.5 and 3 T) was better for the semi-automated method (intraclass correlation coefficient 0.858 for CR and 0.735 for CSA), compared to expert raters (mean coefficient for three expert raters 0.722 for CR and 0.486 for CSA)	
	66 HC	Morphometric parameters (cross-sectional area (CSA), compression ratio (CR), solidity, and torsion)	No morphometric metric showed the discriminative power to distinguish between NMDC and DCM	

AUC, area under the curve; CR, compression ratio; CSA, cross-sectional area; FA, fractional anisotropy; MD, mean diffusivity; AD, axial diffusivity; RD, radial diffusivity; f1, primary partial volume fraction (anisotropic compartment of ball-and-sticks model); d, ball-and-sticks model diffusivity; MTR, magnetic transfer ratio; ^1^H-MRS, single-voxel magnetic resonance spectroscopy; WM, white matter; GM, gray matter; mJOA, modified Japanese Orthopaedic Association scale; tNAA, total N-acetylaspartate.

## Data Availability

Not applicable.
